# Ethnopharmacobotanical study on the medicinal plants used by herbalists in Sulaymaniyah Province, Kurdistan, Iraq

**DOI:** 10.1186/s13002-016-0081-3

**Published:** 2016-01-28

**Authors:** Hiwa M. Ahmed

**Affiliations:** Department of Agricultural Extension, Bakrajo Agricultural Technical Institute, Sulaimani Polytechnic University, Kurdistan, Iraq

**Keywords:** Ethnobotany, Medicinal plants, Kurdistan, Ethnopharmacology, Silemani

## Abstract

**Background:**

Medicinal plants still play an important role in the Kurdish community. Sulaymaniyah Province in South Kurdistan (Iraq) has a great diversity of plants, including medicinal plants, yet very few scattered ethnobotanical studies conducted in Kurdistan are available in the scientific literature. Thus the study of Kurdish ethnobotany may be crucial for understanding local medicinal plant uses and their relationships to surrounding areas. Therefore, the objective of this investigation was to document traditional medicinal plant uses among healers of southern Kurdistan.

**Methods:**

An ethnobotanical survey was conducted to document traditional knowledge on medicinal plants uses among traditional healers in the Province of Sulaymaniyah during 2014 and 2015. The data were collected by interviewing 45 traditional healers (36 males and 9 females between the ages of 25 and 80 years) who retain traditional knowledge on medicinal plants. Furthermore, the use value (UV) of taxa was determined and informant consensus factor (ICF) was calculated for the medicinal plants included in the study. Further analysis was carried out to compare the field data with the Kurdish ethnobotanical literature.

**Results:**

The present study found a total of sixty-six plant species, belonging to sixty-three genera within thirty-four plant families, used to treat ninghty-nine different types of ailments and diseases. The most important family was Lamiaceae (7 species), followed by Apiaceae, Asteraceae, and Fabaceae (6 species each). The most frequently used parts were leaves (46 %), followed by flowers (15 %), and seeds (10 %). The most common preparation method was decoction (68 %), whereas few taxa were consumed as a vegetable (13 %) or ingested in powder form (10 %). The respiratory issues category had the highest ICF value (0.68), followed by inflammations and women’s diseases (0.58 and 0.54, respectively). The highest UVs were recorded for the species *Zingiber officinale* (0.48), *Matricaria chamomilla* (0.37), *Adiantum capillus-veneris* (0.31), *Thymus vulgaris* (0.31) and *Pimpinella anisum* (0.31).

A comparison with previous ethnobotanical studies conducted in Kurdistan (especially within the territory of present-day Turkey) and surrounding areas showed that several medicinal plant reports recorded in the current investigation are new to Kurdish ethnomedicine, and that they have possibly been influenced by other scholarly medical traditions.

**Conclusions:**

The present study demonstrates that the area is rich in medicinal plant knowledge. The information reported by the traditional healers of this region is invaluable for further research in the field of cross-cultural ethnobotany and ethnopharmacology.

## Background

Medicinal plants have been prescribed and used widely for thousands of years to treat various disorders and ailments in traditional herbal medicine systems all over the world [1] and have considerable importance in international trade today [2]. There is an increasing demand for the utilization of medicinal plants for providing primary health care to populations, as they are extensively available and inexpensive [3]. In developing countries, approximately 80 % of the native inhabitants still rely on traditional medicine, mainly based on phytotherapy, for their primary health care [4]. The use of plants as medicine is as old as the history of mankind [5, 6]. Many countries throughout the world, including Iraq [5–7], have their own traditional systems of healing and depend on local folk remedies and Traditional Medicine to meet their needs and treat different diseases. The WHO Traditional Medicine (TM) Strategy 2014–2023 stated that traditional treatments, traditional practitioners and herbal medicines are the main source of health care, if not the only source, for many millions of people [3]. The evaluation of these products and ensuring their safety and efficacy via registration and regulation are major challenges. Recognition of their clinical, pharmaceutical and economic value is still growing, although this varies widely between countries [2]. Nowadays, medicinal plants are not only used as regional and traditional treatments but also registered as official medicines that are verified with pharmacopoeias [8]. Medicinal plants play a major role in pharmacological research and drug development, not only when plant constituents are exploited directly as therapeutic agents, but also as starting materials for the synthesis of drugs or as models for pharmacologically active compounds [2, 9, 10]. In many developing countries traditional medicinal knowledge and practices have not been adequately studied, exploited or documented [11]. These traditional knowledge systems, either lost or transmitted orally from one generation to the next among traditional health practitioners, are in danger due to poor relations between older and younger generations [4, 5, 12]. It has been estimated that about 35,000 to 70,000 plant species are utilized for medicinal purposes globally, of which 6,500 species belong to Asia [13]. Iraq is well known for its great variation in wild plants due to the countries geographical diversity and variable climatic conditions, especially Kurdistan in which Sulaymaniyah Province is located. Traditional medicine in Iraq can be traced back to the Sumerian period (3000–1970 B.C.), and then to the Babylonian and Assyrian periods (1970–589 B.C.) [14]. Hopper and Field (1973) also reported on the useful plants and drugs of Iran and Iraq [15]. Sulaymaniyah boasts a great diversity of plant species given the regions climatic variation and diverse ecological habitats, such as mountains, hills, plains, valleys, and lakes. The different ways of life and rich culture in districts of Sulaymaniyah Province have led to a diverse local health care system. This traditional medicine system depends on the knowledge and practical experience of each individual healer with regard to diagnosing and treating ailments using natural materials. As far as the author is aware, this is the first ethnobotanical study of medicinal plants conducted in Sulaymaniyah Province, Kurdistan, Iraq. The primary objective of this study, therefore, is to identify and document the medicinal plants and associated ethnobotanical knowledge of the local people. It is hoped that these plants will be further studied in order to investigate their phytochemistry and pharmacology.

## Methods

### Study area

**Fig. 1:**
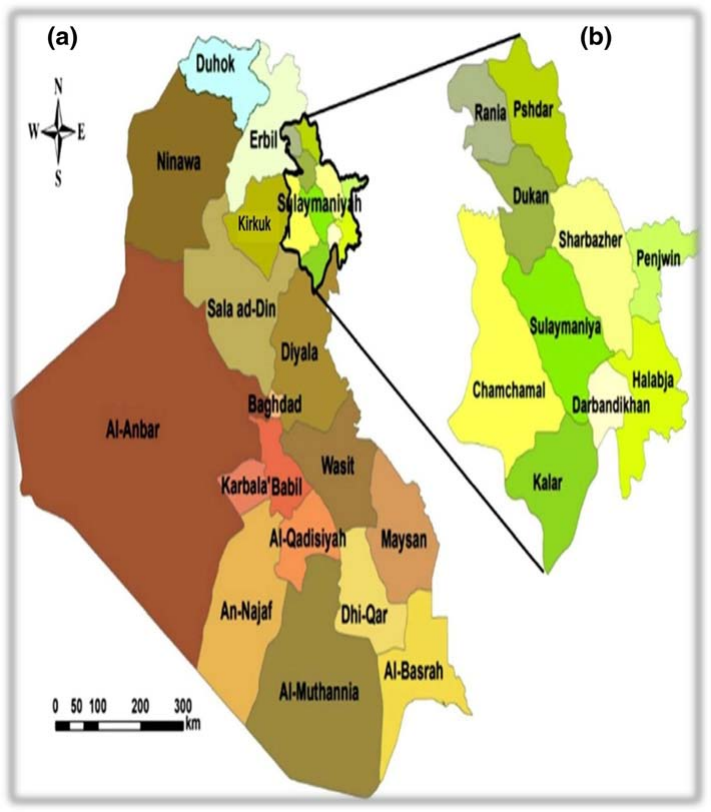
**a** Map of Iraq, **b** Map of the Sulaymaniyah province, where the filed study was conducted

Sulaymaniyah Province (Silêmanî in Kurdish), which is located in southern Kurdistan (northeastern Iraq), is the largest Governorate in Iraqi Kurdistan (Kurdistan Regional Government). The Governorate consists of ten districts, including Rania, Pshdar, Dukan, Sharbazher, Sulaymaniyah, Penjwin, Chamchamal, Halabja, Darbandikhan, Kalar, and borders the country of Iran to the east, and the Iraqi provinces of Erbil, Kirkuk, Salah Al-Din, and Diyala to the North, West, and South respectively. The geographic coordinates (latitude and longitude) of Sulaymaniyah city, the capital of Sulaymaniyah Governorate, are 35°33′40″N and 45°26′14″E, and elevation is about 830 m above sea level. The total area of Sulaymaniyah Province is 17,023 km^2^ [16, 17]. Figure 1 shows the location of the study area including the districts of Sulaymaniyah Province. Sulaymaniyah’s economy today relies on tourism, agriculture, the oil industry and a number of small factories, most of which are involved in the building trade [16, 18]. The province features the fertile plains of Sharazur (Halabja) and Bitwen (Rania), which give way to hills and the Zagros mountain range in the northeast. The climate of Sulaymaniyah is typical for the region as the summer months (June-August) are dry and hot with an average temperature of 31.5 (°C). The winter (December-February) is much colder, wetter and windier, with occasional snowfall and an average temperature of 7.6 (°C). Rainfall, which averages about 400–600 mm per annum [16], starts in October with light storms and intensifies during the month of November and then continues until May.

### Population

Sulaymaniyah, which is officially recognized as the cultural capital of South Kurdistan, has a population of approximately 1.893 million people [16, 17]. In addition, there are almost 180,000 internally displaced persons from the provinces of Anbar, Ninawa, Diyala and Salah Al-Din as a result of generalized violence and armed conflicts, as well as 30,000 Syrian refugees, for whom the Governorate represents a safe haven. The majority of inhabitants in Sulaymaniyah Province are ethnic Sunni Muslim Kurds, but the Governorate is also home to Shiite Kurds and a number of Chaldean Christian communities. The inhabitants mostly speak Sorani Kurdish dialects with a minority of people who speak Hawrami (sub-dialect of Gorani) in some villages of Halabja District [16, 18]. Although the modern health care system is easily accessible everywhere, many people still believe in alternative and complementary medicines including traditional medicine. Therefore, traditional healers continue to play a significant role in local communities.

### Data collection

An ethnobotanical survey was conducted to collect data and document traditional knowledge on the medicinal plants in the province of Sulaymaniyah during 2014–2015. The survey was carried out via semi-structured interviews of traditional healers who have traditional knowledge concerning medicinal plants, adapted from the methods of [4, 6, 19]. The information recorded during the survey included the names and ages of the informants, local names of utilized plants, plant parts used, preparation procedures, method of administration, ailments treated, and duration of treatments (Appendix 1). The healers were chosen randomly among those living in the different districts of the Sulaymaniyah Province. In total, 45 informants, including 36 males and 9 females, between the ages of 25 and 80 years were interviewed in their local language (Kurdish Sorani). For ethical considerations connected to fieldwork, prior to conduct the interviews, permission and ethical approval were obtained from the Department of Agricultural Extension, Bakrajo Agricultural Technical Institute and Sulaimani Polytechnic University. Prior Informed Consent was always verbally obtained before each interview.

### Demographic characteristics of participants

Details of the informants were ascertained and recorded via face-to-face interviews. The majority of informants were healers who practice herbal medicine; a few (five) were farmers or sellers of local products. Demographic details of the informants are provided in Table 1. The majority of informants interviewed were above 40 years of age (~62 %), and males (80 %) greatly outnumbered females (20 %). Although all traditional healers claimed to be experts in traditional medicine [14, 20], only 5 % of them were licensed to dispense herbal medicine. Just over 30 % of informants lacked even primary school education, which may be related to the fact that traditional healing not only uses plants, but also involves rituals and spiritual aspects as part of the therapy [21].

**Table 1:** Demographic details of the interviewed informants

Category	Subcategory	% of informants
Gender	Male	80 %
	Female	20 %
Age	20–30 years	4 %
	31–40 years	33 %
	41–50 years	16 %
	51–60 years	18 %
	61–70 years	22 %
	71 ≥ years	7 %
Education	None	31 %
	Primary	33 %
	Secondary	25 %
	Tertiary	11 %

### Botanical identification

The collected plant specimens were prepared and processed according to the plant taxonomic method [22]. All reported medicinal species were identified with the help of the available literature and Flora of Iraq. The medicinal plant reports of a single plant were accepted as valid only if it was mentioned by at least three independent informants. Voucher specimens, collected from the wild, dried, and assigned a code, were deposited at the Herbarium at Bakrajo Agricultural Technical Institute, Sulaimani Polytechnic University. In this study, scientific names of plant species were checked for accuracy according to The Plant List database (www.plantlist.org).

### Statistical analysis

The information obtained during the interviews was statistically analyzed using Microsoft Office Excel software (2010). On the basis of the data given by the informants in the study area, all the reported ailments were classified into 17 categories and the use value (UV) of taxa was determined. In addition, the informant consensus factor (ICF) was calculated for all medicinal plants included in the study. Further analysis was carried out to compare the current results with previous studies [5, 8, 11, 12, 14, 19, 23–37] conducted in the Kurdish lands of Iraq, Iran, and Turkey in order to identify new medicinal properties of plant species which have not been reported before.

#### Informant consensus factor (ICF)

Informant consensus factor (ICF), which was performed for each category of disease to establish the homogeneity of the information obtained from informants, was calculated according to the formula [38]:

$$ \mathrm{I}\mathrm{C}\mathrm{F} = \mathrm{N}\mathrm{u}\mathrm{r}\ \hbox{--}\ \mathrm{N}\mathrm{t}\ /\ \left(\mathrm{N}\mathrm{u}\mathrm{r}\ \hbox{--}\ 1\right) $$

where, Nur indicates the number of use citations from informants for a particular plant-use category, and Nt refers to the number of taxa or species utilized by all informants for that specific plant use category. ICF values range between 0 and 1, where 1 indicates the highest level of informant consent and 0 the lowest.

#### Use value (UV)

Use value, a quantitative method that demonstrates the relative importance of plant species known locally, was also evaluated according to the following formula [38]:

$$ \mathrm{U}\mathrm{V}\mathrm{i} = {\displaystyle \sum \mathrm{U}\mathrm{i}/\mathrm{N}\mathrm{i}} $$

where, UVi refers to the use value of a species, Ui to the number of citations per specific plant species, and Ni to the number of informants. A high use value indicates the potential importance of the plant species reported.

## Results and discussion

### Medicinal plant diversity, growth habit and plant parts used

**Fig. 2:**
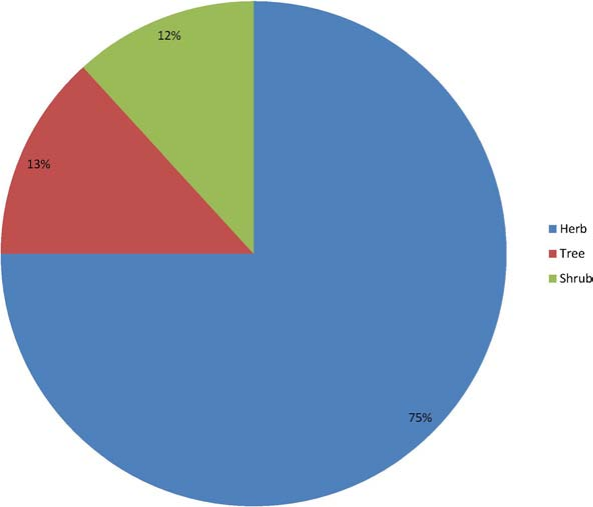
Percentage of the recorded plants according to plant type

**Fig. 3:**
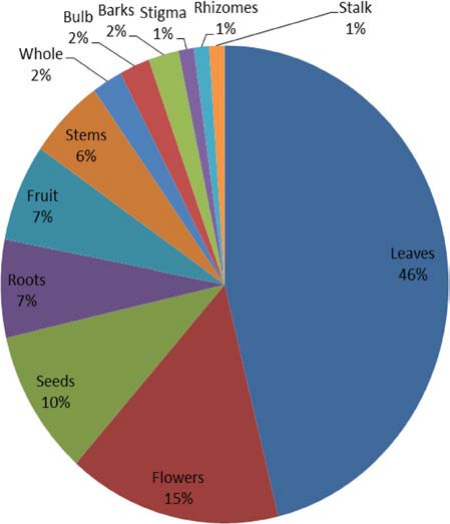
Medicinal plant parts and their percentages

The present study found 66 plant species, belonging to 63 genera within 34 plant families, used to treat 99 different types of ailments and diseases in Sulaymaniyah Province. Table 2 lists the utilized medicinal plant species arranged in alphabetical order by scientific name, family name, English name, Kurdish name, parts used, mode of administration, preparation, medicinal uses and Use Value. The results revealed that the taxonomic family with the greatest number of utilized plants was Lamiaceae (7 species), followed closely by Apiaceae, Asteraceae and Fabaceae (6 species each), and then Rosaceae (4 species) and Malvaceae (3 species). The remaining plant families were represented by only one or two species. This indicates the widespread importance of the abovementioned families in the study area. These results are in general agreement with previous investigations which indicated that the most prominent family was Fabaceae [20, 39, 40]. Figure 2 indicates that herbs (75 %) were the most abundant plants utilized by traditional healers to treat various disorders and ailments in local areas of Sulaymaniyah Province, followed by trees (13 %) and shrub species (12 %). The leaves of medicinal plants (Fig. 3) were the most frequently used parts (46 %) in herbal drugs to cure diseases; however, many other less important plant parts were also employed: flowers (15 %), seeds (10 %), roots (7 %), fruits (7 %), stems (6 %), whole plants (2 %), bulbs (2 %) and barks (2 %), as well as stalks, rhizomes, and stigmas (1 % each). These results are in accordance with what found in studies conducted in other parts of the world [1, 12, 41, 42], which reported the predominant use of plant leaves followed by flowers, fruits and stems. On the other hand, a study conducted in India [20] indicated that roots were the most commonly utilized parts followed by leaves. This discrepancy is probably due to the diversity of plants, weather conditions, and chemical compounds present in the plant parts between the various study areas. As indicated in Table 2, in which we reported the most common preparation methods for various treatments, with instructions on how to administer them, as well as the recommended doses, the most popular preparation was the decoction (nearly 68 %), followed by the use of the plant as a vegetable (13 %), or ingested as a powder (10 %) or hydrodistillation (5 %). Similarly [13] reported that in Iran the most common mode of preparation was decoction (44 %), followed by infusion (21 %), poultice (15 %), oral applications (13 %) and hydrodistillation (7 %).

**Table 2:** Medicinal plants quoted by traditional healers in the study area

Scientific plant name and voucher number	Botanical family name	English name	Kurdish name	Parts used	Mode of administration	Preparation	Local medicinal uses	UV
*Adiantum capillus-veneris* L. *KUR001*	Pteridaceae	Maidenhair fern	Gya qeiteran	Leaves, stalks,	In	Decoction	Asthma, cough, carminative, diarrhea, kidney stones, warts, bladder diseases.	0.31
*Apium graveolens* L. *KUR002*	Apiaceae	Celery	Kerewz	Seeds, leaves	In	Vegetable	Anemia, colon problems.	0.04
*Anethum graveolens* L. KUR003	Apiaceae	Dill	Shwit	Leaves	In	Flavoring, Vegetable	Stomachic, kidney and liver problems, back and arthritis pain, blood cholesterol.	0.15
*Althaea officinalis* L*. KUR004*	Malvaceae	Marsh mallow	Gwle hêro	Leaves	Ex/in	Poultice, decoction	Burns, cough, chest inflammation.	0.13
*Artemisia absinthium* L*.* *KUR005*	Asteraceae	Sea wormwood	Toleke marane	Leaves, flowers	In	Decoction	Anemia, obesity, abdominal pain.	0.06
*Allium sativum* L. *KUR006*	Amaryllidaceae	Garlic	Sir	Bulb	Ex /in	Decoction	Anti-dandruff, intestinal worms, stimulant, blood circulation, rheumatism, cancer, cholera, alopecia areata, tuberculosis, plague.	0.24
*Allium roseum* L. *KUR007*	Amaryllidaceae	Rosy garlic	Gêlaxe	Leaves	In	Decoction, squash	Abdominal and duodenal pain, headache.	0.06
*Aloe vera* (L.) Burm.f. *KUR008*	Xanthorrhoeaceae	Aloe vera	Aloi vira	Leaves	Ex	Decoction	Headache, toothache, indigestion.	0.06
*Arum maculatum* L. *KUR009*	Araceae	Lords-and-ladies	Kardû	Leaves	In	Decoction	Intestinal worms.	0.02
*Borago officinalis* L*. KUR010*	Boraginaceae	Borage	Gozrwan	Leaves, flowers, stems	In	Decoction	Cough, hoarseness, asthma, bronchitis, abdominal pain.	0.11
*Curcuma longa* L. *KUR011*	Zingiberaceae	Turmeric	Zerdeçêwe	Leaves, rhizomes	Ex/in	Decoction	Facial massage, arthritis pain, fat-burning, spice, antiviral and anticancer agent.	0.13
*Citrullus colocynthis* (L.) Schrad*. KUR012*	Cucurbitaceae	Colocynth	Kaleke Marane	Leaves, fruits, seeds	In	Powder, vegetable	Constipation, arthritis pain.	0.04
*Cichorium intybus* L. *KUR013*	Asteraceae	Chicory	Çeqçeqe	Roots, leaves,	In	Decoction	Constipation, blood cholesterol, anemia, colon problems, urinary system problems, prostate problems, skin sensitivity.	0.20
*Cinnamomum cassia* (Nees & T.Nees) J.Presl *KUR014*	Lauraceae	Cinnamon	Darçin	Bark	In	Powder	Stimulant, cough, stress, tuberculosis, clear arteries, improve kidney function, diabetes.	0.15
*Cirsium vulgare* (Savi) Ten. *KUR015*	Asteraceae	Spear thistle	Kingr	Stems	In	Decoction	Inflammation, urinary and digestion problems.	0.06
*Cucumis melo* var*.* flexuosus (L.) Naudin. *KUR016*	Cucurbitaceae	Snake cucumber	Trozi	Stems	In	Powder	Stomach pain, colon problems, diabetes, diarrhea, intestinal inflammation.	0.08
*Crocus sativus* L. *KUR017*	Iridaceae	Saffron	Za'feran	Stigmas	In	Spice	Headache, depression, boost brainpower and sexual function.	0.13
*Ceratonia siliqua* L. *KUR018*	Fabaceae	Carob tree	Xrnûk	Fruits	In	Vegetable	Abdominal pain, diarrhea.	0.04
*Crataegus azarolus* L*. KUR019*	Rosaceae	Hawthorn	Goizh	Leaves	In	Decoction	Kidney and bladder inflammation.	0.04
*Carum carvi* L. *KUR020*	Apiaceae	Caraway	Zire	Seeds	In	Decoction, squash	Immunity, antioxidant, anemia, sleeplessness, indigestion, increasing breast milk.	0.13
*Carlina acaulis* L. *KUR021*	Asteraceae	Stemless carline thistle	Çawbazele	Roots	In	Decoction	Diuretic.	0.02
*Daucus carota* L*.* *KUR022*	Apiaceae	Carrot	Gêzer	Roots	In	Vegetable, squash	Gastric ulcer, bacterial gastroenteritis, diabetes, intestinal worms, eye problems.	0.11
*Eremurus spectabilis* M.Bieb. *KUR023*	Liliaceae	Foxtail lily	Xwzhe	Leaves	In	Decoction	Arthritis, intestinal worms, sedation.	0.06
*Fumaria officinalis* L*. KUR024*	Papaveraceae	Common fumitory	Shatere	Whole	Ex	Powder	Mange.	0.02
*Glycyrrhiza glabra* L. *KUR025*	Fabaceae	Licorice	Belek	Roots	In	Decoction, powder	Gastric ulcer, cough, rheumatism, oral herpes, liver cyrrhosis, abdominal injury.	0.26
*Hibiscus sabdariffa* L*. KUR026*	Malvaceae	Roselle	Çai trsh	Flowers	In	Decoction	Hypertension.	0.02
*Lavandula angustifolia* Mill*. KUR027*	Lamiaceae	Lavender	Sharê'ne romi	Leaves, flowers	In	Decoction	Intestinal worms, antispasmodic.	0.04
*Linum usitatissimum* L*. KUR028*	Linaceae	Flax	Ketani swr	Leaves	In	Powder	Blood cholesterol, rheumatism, colon problems, antispasmodic, skin burns, gallstones, thyroid problems.	0.15
*Lactuca sativa* L*.* *KUR029*	Asteraceae	Lettuce	Kahû	Leaves	In	Vegetable	Kidney stones.	0.02
*Mentha spicata* L*.* *KUR030*	Lamiaceae	Mint	Ping	Leaves, flowers	Ex/in	Hydrodistilled, powder, decoction	Headache, hair tonic, spice, antispasmodic, hiccups, diarrhea, cough, skin diseases, leg pain.	0.22
*Mentha piperita* L*.* *KUR031*	Lamiaceae	Peppermint	Pingi Kêwi	Stems, leaves, flowers	Ex/in	Decoction	Carminative, thoracic pain, indigestion, skin sensitivity, appetizing, colon problems, stomach pain, carminative.	0.26
*Malva parviflora* L. *KUR032*	Malvaceae	Cheeseweed	Toleke	Leaves	Ex	Decoction	Hair loss, abdominal pain, diarrhea.	0.06
*Matricaria chamomilla* L. *KUR033*	Compositae	Wild chamomile	Beibûn	Leaves, flowers	In	Decoction	Hypertension, stomach inflammation, blood circulation, kidney stones, intestinal worms, cough, anxiety, diuretic, headache, abdominal pain, hair loss, sore throat.	0.37
*Medicago sativa* L. *KUR034*	Fabaceae	Alfalfa	Wênge	Stems, leaves, flowers	In	Decoction	Osteomalacia.	0.02
*Nasturtium officinale* R.Br. *KUR035*	Brassicaceae	Watercress	Kûzele	Root, stems	Ex	Decoction	Rheumatism and bone diseases.	0.04
*Nonea versicolor* (Stev.) Sweet *KUR036*	Boraginaceae	Rose monkswort	Çawe pishile	Flowers	Ex	Decoction	Hair loss, skin disease.	0.04
*Nigella sativa* L. *KUR037*	Ranunculaceae	Black cumin	Reshke	Seeds	Ex/in	Hydrodistilled, powder	Eye problems, joint pain and inflammation, chest pain, diabetes, headache.	0.22
*Ocimum basilicum* L. *KUR038*	Lamiaceae	Basil	Rêh'n	Leaves	In	Vegetable	Headache, cold, bad breath, skin, cancer, quit smoking.	0.13
*Olea europaea* L. *KUR039*	Oleaceae	Olives	Zeitûn	Leaves	In	Decoction	Diabetes.	0.02
*Pimpinella anisum* L*. KUR040*	Apiaceae	Anise	Razyane	Seeds, leaves	In	Decoction, powder	Menopause (menstruation), diarrhea (particularly in children), increasing breast milk, ascaris worms, stomachic, appetizing sleepless, colon.	0.31
*Petroselinum crispum* (Mill.) Fuss *KUR041*	Apiaceae	Parsley	Me'denûs	Leaves	Ex/in	Decoction, vegetable	Sexual problems, facial massage, diuretic, appetizing	0.08
*Prunus dulcis* (Mill.) D.A.Webb *KUR042*	Rosaceae	Almond	Badam	Seeds	Ex /in	Hydrodistilled	Hair loss, thoracic inflammation, cough, kidney stones, cold.	0.11
*Pelargonium roseum* Willd*.* *KUR043*	Geraniaceae	Rose geranium	Gwl 'enber	Leaves	Ex	Powder, inhalation	Headache, hemorrhoids, burns.	0.60
*Plantago lanceolata* L*. KUR044*	Plantaginaceae	Ribwort Plantain	Gwê berxe	Leaves	Ex	Powder	Injury treatment, mange.	0.06
*Quercus infectoria* G.Olivier*. KUR045*	Fagaceae	Oak apple	Mazû	Fruits	Ex	powder	Burns.	0.02
*Ranunculus ficaria* L*. KUR046*	Ranunculaceae	Lesser celandine	Gya hengiroke	Leaves, flowers	In	Decoction	Hemorrhoids, arthritis.	0.02
*Ricinus communis* L. *KUR047*	Euphorbiaceae	Castor oil plant	Gerçek	Leaves, fruits	Ex/in	Poultice, hydrodistilled,	Skin diseases, colon problems, hair loss, thoracic pain, constipation, warts.	0.15
*Rosa* spp. *KUR048*	Rosaceae	Rose	Gûle bax	Leaves	In	Decoction	Constipation, abdominal pain.	0.06
*Rosa canina* L. *KUR049*	Rosaceae	Dog rose	Shilan	Flowers, fruits	In	Decoction	Diuretic, blood cell disorders, sedation.	0.06
*Rosmarinus officinalis* L. *KUR050*	Lamiaceae	Rosemary	Rozmêri	Leaves	In	Decoction	Headache, blood circulation, improves memory.	0.06
*Raphanus sativus* L*.* *KUR051*	Brassicaceae	Radish	Tûr	Leaves, bulbs, seeds	In	Vegetable, hydrodistilled	Kidney stones, gallstones, increasing breast milk, diuretic.	0.08
*Rheum ribes* L. *KUR052*	Polygonaceae	Rhubarb	Rêwas	Roots	In	Decoction, squash	Diabetes.	0.02
*Syzygium aromaticum* L*.* *KUR053*	Myrtaceae	Clove	Mêxek	Flowers	In	Decoction, powder, hydrodistilled	Indigestion, stress, insomnia, carminative, toothache.	0.28
*Spinacia oleracea* L. *KUR054*	Amaranthaceae	Spinach	Spênax	Leaves	In	Decoction	Weight loss, phlegm, rickets, bad breath, tuberculosis.	0.11
*Salvia officinalis* L. *KUR055*	Lamiaceae	Sage	Gûle meryem	Leaves, flowers	In	Decoction	Infertility, menopause.	0.04
*Senna alexandrina* Mill*. KUR056*	Fabaceae	Egyptian senna	Sinemeki	Leaves	In	Decoction	Constipation.	0.02
*Salix alba* L. *KUR057*	Salicaceae	White willow	Dar bi spi	Leaves, barks	In	Decoction	Diabetes, cold, blood circulation, headache.	0.11
*Trifolium alexandrinum* L. *KUR058*	Fabaceae	Clover	Sêpere	Leaves	In	Decoction	Colic.	0.02
*Taraxacum officinale* F.H. Wigg *KUR059*	Asteraceae	Dandelion	Gwl xeberhên	Leaves	In	Decoction	Tuberculosis.	0.02
*Trigonella foenum-graecum* L. *KUR060*	Fabaceae	Fenugreek	Shmli	Seeds	In	Powder, decoction	Anemia, diabetes, injures, sore throat, rheumatism, cough.	0.20
*Tribulus terrestris* L. *KUR061*	Zygophyllaceae	Puncture vine	Peikwl	Whole	In	Decoction	Kidney and urinary problems, enhance sexual performance.	0.11
*Thymus vulgaris* L*.* *KUR062*	Lamiaceae	Thyme	Jatre	Seeds, leaves	Ex/in	Decoction	Anti-dandruff, spice, chest pain, colon problems, asthma, diarrhea, blood cholesterol, urinary system problems, menopause.	0.31
*Urtica dioica* L. *KUR063*	Urticaceae	Stinging nettle	Gezne zerdê	Leaves	In	Decoction	Anemia.	0.02
*Viola odorata* L*.* *KUR064*	Violaceae	Sweet violet	Gwle wenewshe	Leaves, flowers	In	Decoction	Diarrhea, digestive problems, skin disorders, typhoid disease, gum disease.	0.11
*Ziziphus jujube* Mill. *KUR065*	Rhamnaceae	Jujube	'enab	Fruits	In	Decoction	Sore throat and asthma.	0.04
*Zingiber officinale* Roscoe *KUR066*	Zingiberaceae	Ginger	Zenjefil	Roots	In	Hydrodistilled, powder, decoction	Asthma, cough, cold, weight loss, chest pain, spice, back pain, hypertension, facial massage, bronchitis, stomach inflammation, nausea, colon problems, diabetes, stimulant.	0.48

### Ailments treated by plants

The traditional knowledge of phytotherapy of this province provides excellent results in the treatment of 99 different types of ailments and diseases in humans (Table 3). On the basis of the knowledge provided by informants the reported ailments were grouped into 17 categories including respiratory issues, inflammations, digestive system disorders, microbial infections, blood problems, diabetes/obesity/weight loss, pain, central nervous system issues, dermatological concerns, kidney problems, rheumatic disorders, women’s diseases, cancer/hoarseness/fat-burning, eye diseases, liver diseases, sexual performance, and musculoskeletal and joint diseases. Informant consensus factor was calculated in order to check homogeneity of the information given, and the respiratory issues category was found to have the highest ICF value (0.68) among all ailment categories. This included the medicinal use of the following species as herbal remedies: *Adiantum capillus-veneris*, *Borago officinalis*, *Thymus vulgaris*, *Ziziphus jujuba*, *Zingiber officinale , Ocimmum basilicum Prunus dulcis*, *Salix alba*, *Althaea officinalis*, *Cinnamomum cassia, Glycyrrhiza glabra*, *Mentha spicata*, *Matricaria chamomilla*, *Prunus dulcis*, *Trigonella foenum-graecum*, *Linum usitatissimum*, *Ocimum basilicum*, and *Spinacia oleracea*. The ailment categories with the next highest ICF values were inflammations, women’s diseases, and diabetes/obesity/weight loss at 0.58, 0.54 and 0.54, respectively. These values of ICF probably indicate the importance of use citations for a particular disorder category and can be a basis for further phytochemical and pharmacological investigations of specific taxa [43, 44]. There are no prior investigations that have estimated ICF in the study area, but compared to other regions differences have been reported. In a survey conducted in Nigde/Aladaglar [37], cardiovascular diseases had the highest ICF value (0.86), whereas respiratory diseases were ranked fifth (0.61) on the list. In the study by [36] in Malatya, constipation had the highest ICF score (0.72). In contrast, in the investigation by [35] in Solhan (Bingöl, Turkey) diabetes had the highest ICF value (0.65).

**Table 3:** Categories of ailment and associated informant consensus factor (ICF) values

No.	Category of ailment	Number of taxa	Use citations	ICF
1	Respiratory issues: asthma, cold, cough, thyroid problems, hiccups, bad breath, phlegm, nausea.	25	76	0.68
2	Inflammations: inflammations of prostate, stomach, chest, and thorax, toothache, bronchitis, bacterial gastroenteritis, gum disease.	13	30	0.58
3	Digestive system disorders: diarrhea, hemorrhoids, constipation, intestinal complaints, carminative, colon problems, indigestion, appetizing, ascaris worms, colic.	37	56	0.34
4	Microbial infections: typhoid, cholera, tuberculosis, plague, antiviral agent, immunity, antioxidants, oral herpes.	11	13	0.16
5	Blood problems: cholesterol, hypertension, anemia, stimulant, circulation, clear arteries, blood cell disorders.	21	38	0.45
6	Diabetes/obesity/weight loss.	12	25	0.54
7	Pain: arthritis, backache, headache, chest pain, abdominal pain, duodenal pain, stomachache, leg pain, thoracic pain, sore throat, joint pain.	43	60	0.28
8	Central nervous system issues: stress, depression, boost brainpower, sleeplessness, insomnia, sedation, anxiety,improve memory	11	12	0.09
9	Dermatological concerns: skin diseases, hair loss, hair tonic, anti-dandruff, burns, wounds, warts, alopecia areata, facial massage, mange.	28	38	0.27
10	Kidney problems: kidney disorders, bladder infections, urinary system problems, diuretic, gastric ulcer, gallstones.	23	36	0.37
11	Rheumatic disorders: rheumatoid arthritis, antispasmodic.	8	13	0.41
12	Women’s diseases: menopause, increasing breast milk.	6	12	0.54
13	Cancer/hoarseness/fat-burning.	5	5	0.00
14	Eye diseases: eye problems.	2	3	0.50
15	Liver diseases.	2	2	0.00
16	Sexual performance: sexual function, infertility.	4	4	0.00
17	Musculoskeletal and joint diseases: osteomalacia, bone diseases, rickets.	3	3	0.00

The high ICF value (0.68) for the respiratory issues category in traditional Kurdish medicine may be due to the fact that the region and in particular big cities have not met sufficient ecological standards. Air quality of the region may also poor due in part to a chemical weapons attacks occurring in the past decades; in addition, in recent years dust storms that have lasted for several days and even a week have hit Kurdistan. These dust storms, which affect a wide geographical area, result in an increase of dust particles in the air and create medical complications among inhabitants, particularly respiratory problems. Use values (UV) indicate the relative importance of plant species among practitioners (Table 2). In this study the highest UV values were recorded for *Zingiber officinale* (0.48), *Matricaria chamomilla* (0.37), *Adiantum capillus-veneris* (0.31), *Thymus vulgaris* (0.31), and *Pimpinella anisum* (0.31). Previous ethnobotanical investigations conducted in Turkish Kurdistan have reported different UV values; for instance, a study conducted in Nigde/Aladaglar recorded the highest use value (0.51) for *Hypericum perforatum* [37]. Conversely, *Armeniaca vulgaris* (0.53), *Urtica dioica* (0.44), and *Mentha spicata* (0.42) were reported to have the highest use values in Malatya [36]. As calculated by [35], in Solhan (Bingöl, Turkey), *Urtica dioica* (0.59), *Malva neglecta* (0.52), and *Rosa canina* (0.50) were reported to be the most important medical plants.

The high use value we recorded for *Zingiber officinale* (0.48) in the study area may be due in part to the fact that Kurdish ethnomedicinal knowledge has likely been influenced by Islamic traditional medicine, as mentioned in the Holy Quran [45] or addressed by Arabic scholarly medical traditions of the Middle East.

In our study we recorded in fact also some species mentioned in the Holy Quran, such as olives, ginger, garlic, basil, and castor oil plant, as well as by Muhammad, the Prophet of Islam (i.e. black cumin, chicory, fenugreek, watercress, celery, colocynth, dill, and hawthorn).

The present study shows that not all the mentioned plants are cultivated locally and most of them grow naturally in the wild. Furthermore, many plant materials pass the borders from neighboring countries without proper inspection and verification by quality control. Although pharmaceuticals are available in local areas, herbal medicine has remained popular in Kurdistan for various historical and cultural reasons. Interestingly, traditional healers mentioned that in cases in which a chemical medicine has no effect on their health, patients sometimes attempt to use herbal medicines as an alternative to chemical drugs with the hope of curing certain disorders and ailments. Therefore, documentation of local folk knowledge through ethnobotanical studies is essential for the conservation and utilization of these medical traditions [41, 46]. Plants as medicine play a significant role in the public health sector worldwide, as many cultures share a strong belief in their ability to cure certain diseases [14].

### Comparison with the Kurdish medical ethnobotany

Previous studies conducted in Kurdistan (Kurdish lands in current Iraq, Iran, and Turkey) reported the exploitation of some medicinal plants, which were also documented in the present study. However, this is the first ethnobotanical study in Sulaymaniyah, and thus 17 plant species and 49 new properties of plant species were found in the present investigation that have never been reported before in Kurdistan and surrounding areas (Table 4), including the use of the following: *Allium roseum* to alleviate abdominal and duodenal pains, and headaches; *Aloe vera* to relieve headaches, toothaches, and indigestion; *Borago officinalis* to treat cough, hoarseness, asthma, bronchitis, and abdominal pain; *Curcuma longa* to alleviate arthritis pain, to burn fat, and as a spice, as well as antiviral and anticancer agents; *Cirsium vulgare* to treat inflammations as well as urinary and digestive tract problems; *Cucumis melo* var. *flexuosus* to treat stomach pain, colon problems, diabetes, diarrhea, and intestinal inflammation; *Ceratonia siliqua* to alleviate abdominal pain and diarrhea; *Lavandula angustifolia* to cure intestinal worms and as an antispasmodic; *Mentha piperita* to treat flatulence, thoracic pain, indigestion, skin sensitivity, colon problems, and stomach pain, as well as an appetizing and carminative; *Pelargonium roseum* to treat headaches, hemorrhoids, and burns; *Ranunculus ficaria* to relieve haemorrhoids and arthritis; *Rosa canina* to treat blood cell disorders, and as a diuretic and sedative; *Raphanus sativus* to cure kidney stones and gallstones, for increasing breast milk, and as a diuretic; *Spinacia oleracea* to treat phlegm, rickets, bad breath, and tuberculosis, as well as for weight loss; *Trifolium alexandrinum* to cure colic; and *Ziziphus jujuba* to alleviate sore throat and asthma.

**Table 4:** New folk medicinal reports for Kurdistan found in the current study

Plant scientific name	New folk medicinal plant reports	Folk medicinal reports previously recorded in the Kurdish ethnobotany
*Adiantum capillus-veneris* L.	Asthma, carminative, diarrhea, warts	Kidney and bladder stones, diuretic, cough [28, 29], abdominal pain [34].
*Apium graveolens* L.	Anemia, colon problems	Diuretic, dysmenorrhea and rheumatism, arthritis, stimulant, carminative, tonic.
*Anethum graveolens* L.	Kidney and liver problems, back and arthritis pain	Carminative for flatulence in children, cough, children’s stomach pain [5], hypercholesterolemia [23, 30].
*Althaea officinalis* L.	Burns, cough, chest inflammation	Irritation and inflammation of the mucous membranes [25].
*Artemisia absinthium* L.	Anemia, obesity, abdominal pain	Diabetes, shortness of breath [30], eczema, coughing, headache, stomach-ache, wound healing [32].
*Allium sativum* L.	Anti-dandruff, intestinal worms, stimulant, rheumatism, cholera, alopecia areata, tuberculosis, plague	Hypertension [23, 26, 29, 35] ringworm [29], common cold, anticancer and antibacterial agents, enhancing immunity, hypercholesterolemia [23].
*Allium roseum* L.	Abdominal and duodenal pain, headache	None.
*Aloe vera* (L.) Burm.f.	Headache, toothache, indigestion	None.
*Arum maculatum* L.	Intestinal worms	Rheumatism [36].
*Borago officinalis* L.	Cough, hoarseness, asthma, bronchitis, abdominal pain	None.
*Curcuma longa* L.	Facial massage, arthritis pain, fat-burning, spice, antiviral and anticancer agents	None.
*Citrullus colocynthis* (L.) Schrad.	Laxative, arthritis pain	Diabetes [12, 28], wounds [12].
*Cichorium intybus* L.	Constipation, blood cholesterol, anemia, colon problems and skin sensitivity	Antihypertensive, prostate problems, stomach ache [30], burns, stomach-ache [32], hemorrhoids, urinary disorders [36].
*Cinnamomum cassia* (Nees & T.Nees) J.Presl	Stimulant, stress, tuberculosis, clear arteries, improves kidney function	Antibacterial, general tonic, diuretic, renal failure, anemia, sexual tonic for men [5] antidiabetic [24, 28], hyperglycemia, cough, flatulence, common cold, and pinworm [28].
*Cirsium vulgare* (Savi) Ten.	Inflammation, urinary and digestive problems	None.
*Cucumis melo* L.	Stomach pain, colon problems, diabetes, diarrhea, intestinal inflammation	None.
*Crocus sativus* L.	Headache, boosting brainpower and sexual function	Sedation [28], metabolism stimulant [5, 28], carminative, fever and depression [5], breezy, tonic for heart and culinary use [12], Infertility [34].
*Ceratonia siliqua* L.	Abdominal pain, diarrhea	None.
*Crataegus azarolus* L.	Bladder inflammation	Antihypertensive, headache [12], anti-diabetic, rheumatism [24, 35], insomnia, joint pain, stress [24, 35], ulcer [35], cardiac disorder [12, 24, 26, 27, 29, 35, 37], vasodilators [19, 27, 29], circulation problems, spasm, sedative, to take out heel spur, nail, bullet [37], renal caculi [34], embolism [35], stomach-ache [35], asthma, hemorrhoids [35].
*Carum carvi* L.	Immunity, antioxidants, anemia, sleeplessness, indigestion, increasing breast milk	Digestive system, stomach pain, flavoring agent and mild laxative [5], appetizing, digestive [27], carminative, abdominal pain [28].
*Carlina acaulis* L.	Diuretic	None.
*Daucus carota* L. ssp. *sativus* (Hoffm.) Schübl. & G.Martens	Gastric ulcer, bacterial gastroenteritis, diabetes, intestinal worms	Eye sight [28].
*Eremurus spectabilis* M.Bieb.	Arthritis, intestinal worms	Rheumatism [27], diabetes, digestive [35], sedative [37].
*Fumaria officinalis* L.	Mange	Tonic, diaphoretic, sporadic stomach and antispasmodic [5], stomach-ache [27], fomentation in painful swellings, stomach pain, febrifuge, blood purifier and antispasmodic [5], dermal discords, wound, eczema [12], hemorrhoids [26, 27], sore [33].
*Glycyrrhiza glabra* L.	Cough, rheumatism, oral herpes, liver cirrhosis	Pneumonia, sour eructations, duodenal inflammation, kidney pain, abdominal pain [28], digestive [29, 36], high cholesterol [29], cardiac disorders, diabetes [30], vaginitis, quit smoking, anti-ulcer, anti-aphthous [34], stomach-ache [36].
*Hibiscus sabdariffa* L.	None	Obesity, restorative, anti-hypertensive, sedative, dyspnea [28].
*Lavandula angustifolia* Mill.	Intestinal worms, antispasmodic	None.
*Linum usitatissimum* L.	Rheumatism, colon problems, antispasmodic, skin burns, gallstones, thyroid problems	Obesity, hypercholesterolemia, perfume, apply on body or clothes, witchcraft, expel lochia [28].
*Lactuca sativa* L.	Kidney stones	Typhoid fever, emollient for skin, hypnotic and narcotic [5].
*Mentha spicata* L.	Headache, strengthen hair, hiccups, diarrhea, cough, skin diseases, leg pain	Colds [19, 26, 27, 29, 33, 35, 36], flu [19, 27, 29, 35, 36], stomach ache [19, 33], antispasmodic [27], vomit [29], food poisoning [2], appetite [35], respiratory problem [35, 36].
*Mentha piperita* L.	Flatulence, thoracic pain, indigestion, skin sensitivity, appetizing, colon problems, stomach pain, carminative	None.
*Malva parviflora* L.	Diarrhea	Pectoral, expectorant, laxative [5, 32], interstitial infection, laxative, sore throat, asthma [12], diuretic, urinary inflammations [19, 27, 34], stomach ache [26], hemorrhoids [27], abscess, hematomas, anti-inflammatory [29, 34], joint pain [29], gastric pain [30, 32], wound healing [30, 32, 34, 36, 37], rheumatism [32], abdominal pain, infertility [34], mastitis, psoriasis, vaginal candidiasis [36], respiratory problems, digestion problems, abortifacient [37], hair loss and constipation [33], anti-inflammatory, infertility, urinary inflammations [35], headache [32].
*Matricaria chamomilla* L.	Hypertension, stomach inflammation, blood circulation, kidney stones, intestinal worms, anxiety, diuretic, headache, throat pain	Wounds, diarrhea [35], cough, tonsillitis, dyspnea [shortness of breath], common cold, asthma, polyps, flatulence, abdominal pain, pharyngitis, black pigments on face, facial rash, acne, skin burns, skin tonic, hair loss, jaundice [28].
*Medicago sativa* L.	Osteomalacia	Tonic and fattening [12], skin bleeding [30], stomach-ache, coagulation, infection [33].
*Nasturtium officinale* R.Br*.*	Rheumatism and bone diseases	Stomachic [12, 35], anti-parasite [12], antidiabetic [24], vaginitis [34], antihypertensive [35].
*Nonea versicolor* (Stev.) Sweet	Hair loss, skin diseases	Snake bite [30].
*Nigella sativa* L.	Eye problems, joint pain and inflammation, chest pain, headache	Repelling gases, antibacterial, antiviral, sexual tonic, enhance memory, tonic and allergy, enhance immune system [23, 28], bronchitis and asthma [23], diabetes, antihypertensive, cancer, pneumonia, tonsillitis, hyperlipidemia, blood circulation, restorative [28], galactogogue, anti-hyperpigmentation, anti-scar [34].
*Ocimum basilicum* L.	Bad breath, skin, cancer, quit smoking	Stomach-ache [29], headache [34], colds and flu [35], abdominal pain [36].
*Olea europaea* L.	None	Diabetes [28].
*Pimpinella anisum* L.	Menopause (menstruation), diarrhea (particularly in children), increasing breast milk, ascaris worms, stomachic, appetizing, sleeplessness.	Carminative, culinary remedy [12, 37], flu, cough, diuretic, analgesic, indigestion, anxiety [23], flatulence [23, 28], asthma, female fertility [ovulatory stimulant], hypochondria, agony, colonitis, antihypertensive, abdominal pain, urolithiasis [28].
*Petroselinum crispum* (Mill.) Fuss	Sexual problems, facial massage, diuretic, appetizing, problem	Kidney stones, mouth sores [29].
*Prunus dulcis* (Mill.) D.A.Webb	Thoracic inflammation, cough, kidney stones, cold	Rheumatoid arthritis, strengthens hair, eye pain, hypercholesterolemia, facial treatment [28].
*Pelargonium roseum* Willd.	Headache, hemorrhoids, burns	None.
*Plantago lanceolata* L.	Mange	Antipyretic [29], wound healing [29, 30, 32–37], diabetes [30], gastric pain [30, 32], hemorrhoids [30, 32, 33], coughs, infections, pains [33], overcoming infertility in women [34], embolism, abscess [35], urinary inflammations [35, 36], expectorant, tonsillitis [36].
*Quercus infectoria* G.Olivier.	None	Oral sores, wounds [28], diabetes [29], burns [34], toothache [36].
*Ricinus communis* L.	Skin diseases, colon problems, thoracic pain, warts	Purgative, strengthens hair [28].
*Rosa* spp.	Constipation, abdominal pain	Indigestion, diuretic, blood cells, sedatives [12], antiseptic [19, 27], diabetes [19, 24, 26, 27, 29, 34], flu [19, 27, 32, 35], antitussive [29, 30], coughing [32], expectorant, kidney stones [35], colds, [19, 27, 29, 30, 32, 35–37], hemorrhoids [35, 36].
*Ranunculus ficaria* L.	Hemorrhoids, arthritis	None.
*Rosa canina* L.	Diuretic, blood cell disorders, sedative	None.
*Rosmarinus officinalis* L.	Headache, blood circulation	Enhances memory and concentration [28].
*Raphanus sativus* L.	Kidney stones, gallstones, increasing breast milk, diuretic	None.
*Rheum ribes* L.	None	Hypertension [12, 23], triglyceride [12], hypoglycemic [23], urinary inflammations [19], digestive, diuretic, constipation, high cholesterol [27], kidney stones [27, 29, 34, 35], diabetes [29, 30, 32, 35, 36], headache [32], rheumatic pain control, anti-diarrhea [34], asthma, cardiac disorder [35].
*Syzygium aromaticum* L.	Indigestion, stress, insomnia, intestinal gas	Toothache, painkiller, headache, asthma, ingredient [28].
*Spinacia oleracea* L.	Weight loss, phlegm, rickets, bad breath, tuberculosis	None.
*Salvia officinalis* L.	None	Regulate menstrual cycle, hypoglycemic, hypercholesterolemia, flatulence, antibacterial, fever [23], Alzheimer’s, cough, digestive, flu, tonsillitis [27], appetite stimulant, female fertility, female aphrodisiac [28], women fertility and infections, cold, anti-fever [12], antidiabetic [24], diabetes disease, cold and flu [26, 29, 36], digestive, tonsillitis [36], antacid [36], sedative, bleeding, cold, diarrhea, sedative, digestive [37].
*Senna alexandrina* Mill.	Constipation	Purgative, obesity, diarrhea, colonitis [28].
*Salix alba* L.	Cold, blood circulation	Anti-fever [12, 37], diabetes [29], toothache [30], headache [34], analgesic [35, 36], infertility [35], sinusitis [35, 36], restorative [37].
*Trifolium alexandrinum* L.	Colic	None.
*Taraxacum officinale* F.H. Wigg	Tuberculosis	Arthralgia, diuretic [27], digestive [29].
*Trigonella foenum-graecum* L.	Anemia, sore throat, rheumatism	Skin inflammation [28], irritable bowel,appetizimg, hypoglycemic, diuretic, stimulate lactation, sexual tonic in women, UTI, renal stones [23], diabetes, hemostatic for diabetics, vulnerary, antihypertensive, infection in body, appetite stimulant, sedative, catarrh, lactation, enhances sperm production, hypercholesterolemia [28].
*Tribulus terrestris* L.	Urinary problems, enhance sexual function	Diarrhea [27, 36], antihypertensive [29], kidney stones [30, 35], dissolves renal caculi, ulcer [34], asthma, cardiac disorder [35], cardiac disorder, hemorrhoids, vasodilators [36].
*Thymus vulgaris* L.	Anti-dandruff, spice, chest pain, colon problems, asthma, diarrhea, blood cholesterol, urinary system problems, menopause	Gingivitis, dyspepsia, appetizing, abdominal cramps, antifungal, anthelmintic, expectorant, tonic, enhance immune system, cystitis and nephritis [23].
*Urtica dioica* L.	Anemia	Arthralgia, colds, flu [19, 27, 32], diabetes [19, 27, 29, 32], throat diseases, painkiller and to reduce blood sugar level [26], rheumatism [27, 30, 32, 35], losing weight [27], bronchitis, cardiovascular disease, cough, respiratory disease, tonsillitis [29] colds [30], cancers [30, 32, 36], stomach ache [32], analgesic, arthritis, digestive, diuretic, genital disorders, hemorrhoids, hepatitis, leptotrichia [35], digestive, diuretic, genital disorders, hemorrhoids [36], urinary system, ulcer, constipation [36].
*Viola odorata* L.	Diarrhea, digestive problems, skin disorders, typhoid disease and gum disease	Gastritis, gastric and kidney pain [30], prostate [36].
*Ziziphus jujuba* Mill	Sore throat and asthma	None.
*Zingiber officinale* Roscoe	Asthma, cold, weight loss, chest pain, spice, back pain, facial massage, bronchitis, stomach inflammation, nausea, blood sugar, stimulant	Hypercholesterolemia, sexual tonic, regulate blood circulation, hemorrhoid, stomach and respiratory problems [23], obesity, flatulence, abdominal pain, cough, tonsillitis, prostate problems, tonsillitis, female fertility, pneumonia, enhances bile secretion, body pain, Malta fever, restorative, rheumatism, blood circulation, sweating, colonitis, antihypertensive [28].

Of the abovementioned species a few ones, and most notably *Eremurus spectabilis* and *Rheum ribes* are prototypical plant species native to Kurdistan.

Interestingly, there are certain mixtures of different medicinal plants that are often used for a variety of purposes, for instance a mixture of *Pimpinella anisum* with *Nigella sativa* is used not only to treat menopause (menstruation), diarrhea (particularly in children), ascaris worms, sleeplessness, and colon problems, but also as an appetizing and stomachic, and for increasing breast milk. Similarly, a combination of *Adiantum capillus-veneris* and *Crataegus azarolus* with *Tribulus terrestris* is used to cure kidney and urinary system problems. Moreover, the current study showed that the folk remedies used by healers in Sulaymaniyah are sometimes mixtures of diverse plants, thus suggesting the influence that scholar medical systems (notably the Arabic and the Persian) may have had in shaping the ethnobotanical traditions we recorded.

For example, a combination of air-dried flowers of *Althaea officinalis* are ground and mixed with a little milk, then applied to burns twice daily. Also, the flowers of this plant are boiled and the decoction is then filtered and drunk after having a meal to treat cough and chest inflammation. In addition, *Zingiber officinale* mixed with powdered corn and oil is used to alleviate back pain. A number of different plants seem to be reported for the first time in this study; these uses need to be investigated pharmacologically to confirm the biological activities that have been claimed for them.

The study area is considered the most famous and important area of Kurdistan, and possibly even all of Iraq, with lofty mountains and scattered flora, many of which are still unexplored from taxonomic and medicinal points of view. Many herb species have been scientifically studied and their pharmacological properties discovered. For example, *Nigella sativa* has been found to possess antidiabetic, anticancer, antimicrobial, anti-inflammatory, and antioxidant properties, as well as act as an immunomodulatory, analgesic, bronchodilator, hepato-protective, renal protective, and gastro-protective, among others [47]. *Salvia officinalis* is another well-known herb which has demonstrated interesting pharmacological properties, such as antioxidant, anti-microbial, anti-inflammatory, analgesic, antipyretic, hemostatic, hypoglycemic and antitumor [48]. Herbalists should be aware of the side effects of the prolonged use of herbal medicines, otherwise it could possibly lead to toxic levels. For instance, as commonly known, the use of *Salvia officinalis* may lead to epileptic seizures, if taken in large quantities [49]. Also, not much is known about the interactions that may occur with either other medicines or food taken at the same time during the treatment period. In recent years, the use of herbal medicine has expanded dramatically in the search of new medical entities or novel lead nuclei with the prospect of managing diverse diseases [9, 10].

## Conclusions

The current study conducted in the Sulaymaniyah Province recorded sixty-six plant species belonging to sixty-three genera distributed across thirty-four families that have been indicated by the interviewed healers to be able to treat nighty-nine human ailments. The current study reported seventeen new medicinal species that had not been previously reported in the Kurdish ethnomedicine. The present study also demonstrates that southern Kurdistan, due to its geographical and cultural diversity, is rich in medicinal plant knowledge that can be important for being considered for treating a wide range of human ailments.

Sulaymaniyah Governorate, as part of the Kurdistan region of Iraq, has a quite well-developed health care system across the region and many people have access to modern medicine even in rural areas. In addition, many people living in villages are strongly linked to cities as their family is often divided into two parts, one part living in the city and the other one in the village. The residents of both rural areas and cities still trust traditional medicine as a path to well-being. Therefore, the patients in Kurdistan could continue to take advantage of these traditional medicines by encouraging scientific research aimed at the bio-evaluation of these invaluable species, leading to the development of local plants-based phytomedicines. Moreover, today medicinal plants face extinction or severe genetic loss due to over-harvesting and exploitation. As a result of this, special attention is urgently required to gather and systematically document this empirical knowledge and to protect and conserve wild medicinal plants, and their habitats, as well as traditional knowledge concerning their use. It is important for all herbalists and those dealing with medicinal plants to save them from extinction as they play an essential role in the public health sector.

These findings suggest that medicinal plants and folk medicines used by healers in Southern Kurdistan may represent a starting point for further comparative cross-cultural ethnobiological research, which may contribute to increase the current knowledge of folk medicinal plants and could lead to the conservation strategies aimed at protecting possible rare plant species. The current research contributed to the existing ethnobotanical literature by identifying a number of new plant uses and their perceived health benefits to humans. Perhaps, more importantly, the results of this study could assist small-scale companies to utilize local plant resources for medicine, as natural products meet the demand of patients, who also in Kurdistan desire less pharmaceuticals; moreover, medicinal plants may provide economic benefits to local communities as well, in an area of the Middle East, which have gone through hard times in the last decades.

## Appendix 1

- Date
- Village/town
- Given name and surname of informant
- Age and gender
- Level of education
- Which kinds of plants do you use to treat different ailments?
- What is the vernacular name of these plants?
- Do you know any other names for these plants?
- How have you learned to recognize them?
- Which plant parts do you use and for what diseases?
- Can you describe the preparation of the remedy in detail?
- Is the remedy internally or externally administered?
- When should the medicine be taken and for how long?
- Where and when did you acquire this knowledge?
